# *N*′-[Bis(benzyl­sulfan­yl)methyl­idene]benzohydrazide

**DOI:** 10.1107/S1600536812019472

**Published:** 2012-05-05

**Authors:** Shahedeh Tayamon, Thahira Begum S. A. Ravoof, Mohamed Ibrahim Mohamed Tahir, Karen A. Crouse, Edward R. T. Tiekink

**Affiliations:** a Department of Chemistry, Universiti Putra Malaysia, 43400 Serdang, Malaysia; b Department of Chemistry, University of Malaya, 50603 Kuala Lumpur, Malaysia

## Abstract

In the title hydrazonodithio­ate, C_21_H_19_N_3_OS_2_, the amide group is twisted out of the plane through the S_2_C=N atoms: the C—N—N—C torsion angle is 139.71 (13)°. The pyridine ring forms dihedral angles of 52.96 (8) and 86.46 (8)° with the phenyl rings, and the latter are approximately orthogonal [dihedral angle = 76.42 (9)°]. Supra­molecular chains sustained by N—H⋯O hydrogen bonds and propagated by glide symmetry along the *c* axis are found in the crystal structure. The chains are consolidated into a three-dimensional architecture by C—H⋯O and C—H⋯N inter­actions.

## Related literature

For background to the coordination chemistry of dithio­carbazate derivatives, see: Tarafder *et al.* (2002 ▶); Ravoof *et al.* (2010 ▶). For related syntheses, see: Ali & Tarafder (1977 ▶); Ali *et al.* (2001 ▶); Manan *et al.* (2012 ▶). For related structures, see: Jasinski *et al.* (2010 ▶); Singh *et al.* (2007 ▶).
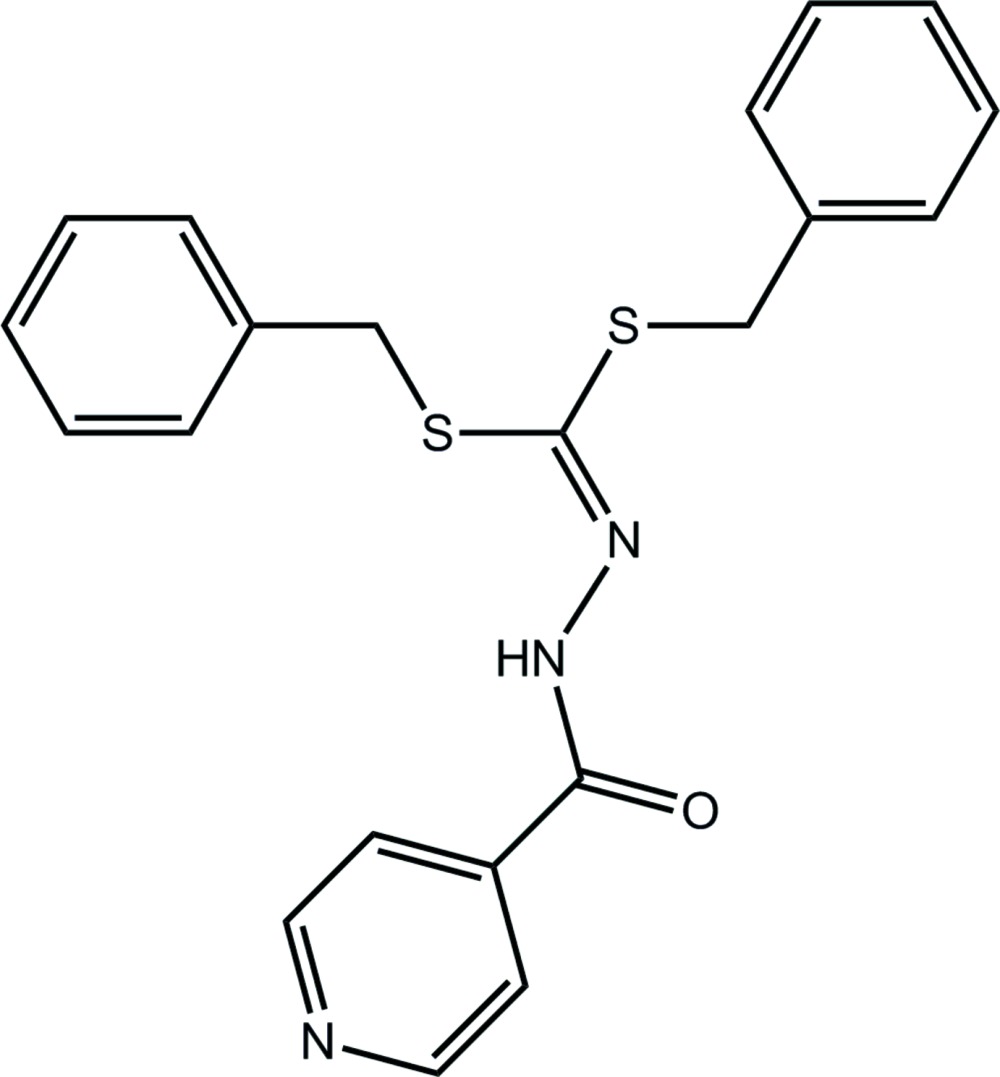


## Experimental

### 

#### Crystal data

C_21_H_19_N_3_OS_2_
*M* *_r_* = 393.51
Monoclinic, 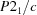

*a* = 11.2593 (4) Å
*b* = 21.2182 (7) Å
*c* = 8.6041 (3) Å
β = 103.678 (3)°
*V* = 1997.24 (12) Å^3^
*Z* = 4
Cu *K*α radiation
μ = 2.54 mm^−1^
*T* = 150 K
0.50 × 0.36 × 0.16 mm

#### Data collection

Agilent Xcaliber Eos Gemini diffractometer
Absorption correction: multi-scan (*CrysAlis PRO*; Agilent, 2011 ▶) *T*_min_ = 0.42, *T*_max_ = 0.67
38338 measured reflections
3867 independent reflections
3715 reflections with *I* > 2σ(*I*)
*R*_int_ = 0.031

#### Refinement

*R*[*F* ^2^ > 2σ(*F* ^2^)] = 0.035
*wR*(*F* ^2^) = 0.097
*S* = 1.03
3867 reflections
247 parameters
H atoms treated by a mixture of independent and constrained refinement
Δρ_max_ = 0.22 e Å^−3^
Δρ_min_ = −0.32 e Å^−3^

### 

Data collection: *CrysAlis PRO* (Agilent, 2011 ▶); cell refinement: *CrysAlis PRO*; data reduction: *CrysAlis PRO*; program(s) used to solve structure: *SHELXS97* (Sheldrick, 2008 ▶); program(s) used to refine structure: *SHELXL97* (Sheldrick, 2008 ▶); molecular graphics: *ORTEP-3* (Farrugia, 1997 ▶) and *DIAMOND* (Brandenburg, 2006 ▶); software used to prepare material for publication: *publCIF* (Westrip, 2010 ▶).

## Supplementary Material

Crystal structure: contains datablock(s) global, I. DOI: 10.1107/S1600536812019472/hb6755sup1.cif

Structure factors: contains datablock(s) I. DOI: 10.1107/S1600536812019472/hb6755Isup2.hkl

Supplementary material file. DOI: 10.1107/S1600536812019472/hb6755Isup3.cml

Additional supplementary materials:  crystallographic information; 3D view; checkCIF report

## Figures and Tables

**Table 1:** Hydrogen-bond geometry (Å, °)

*D*—H⋯*A*	*D*—H	H⋯*A*	*D*⋯*A*	*D*—H⋯*A*
N2—H2N⋯O1^i^	0.864 (17)	1.936 (17)	2.7852 (15)	167.4 (16)
C7—H7⋯N3^ii^	0.95	2.54	3.339 (2)	142
C8—H8⋯N3^iii^	0.95	2.52	3.424 (2)	158
C18—H18⋯O1^i^	0.95	2.53	3.365 (2)	147

Symmetry codes: (i) 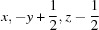; (ii) 
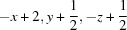
; (iii) 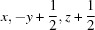.

**Fig. 1.**
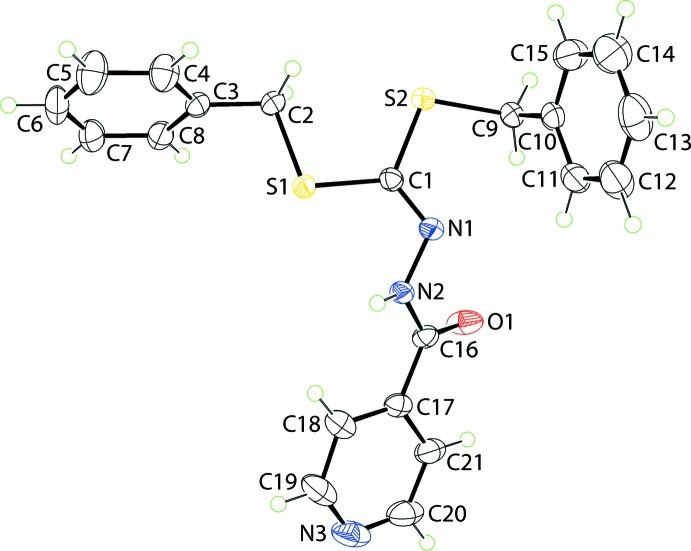
The molecular structure of (I) showing displacement ellipsoids at the 50% probability level.

**Fig. 2.**
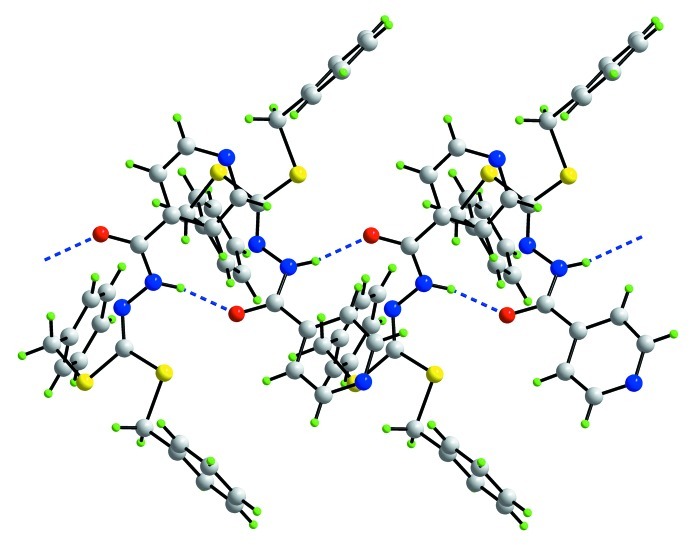
A view of the supramolecular chain in (I) mediated by N—H···O hydrogen bonding, shown as blue dashed lines.

**Fig. 3.**
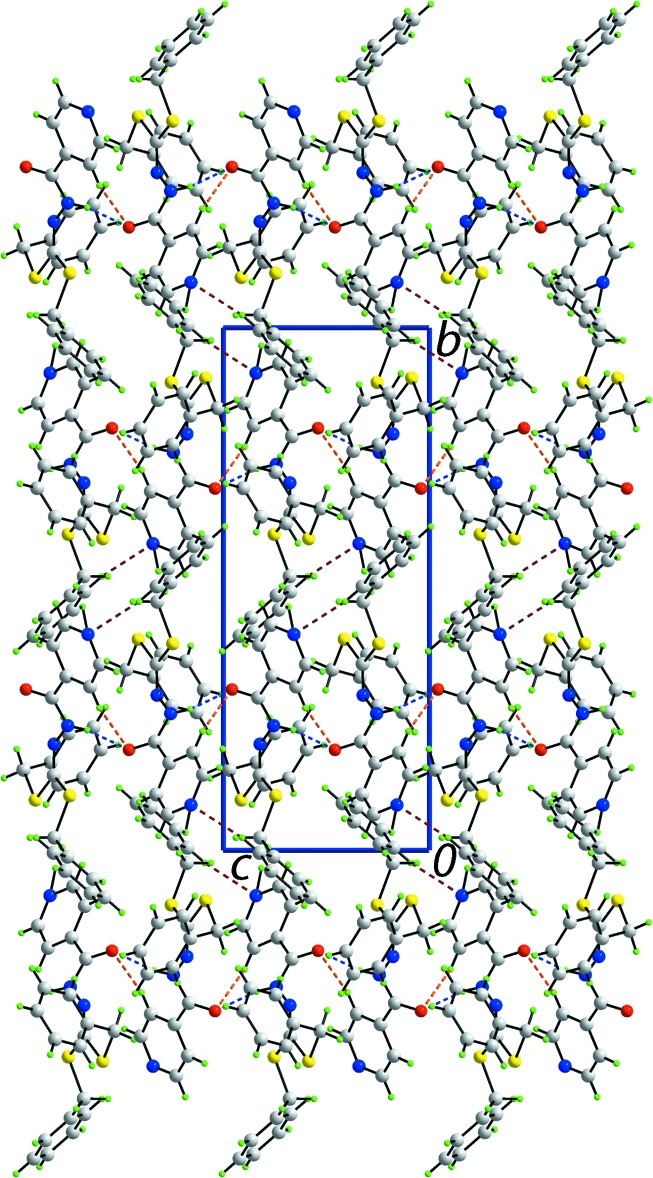
A view in projection down the a axis of the unit-cell contents for (I). The N—H···O, C—H···O and C—H···N interactions are shown as blue orange and brown dashed lines, respectively.

## supplementary crystallographic information

### Comment

Our interest in investigating the coordination properties of ligands containing the H—N—C═S moiety (Tarafder *et al.*, 2002; Ravoof *et al.*, 2010) and our desire to expand the study of this class of biologically important compounds has lead us to synthesize a series of related ligands (Ali *et al.*, 2001; Manan *et al.*, 2012). The title compound *N*'-bis(benzylsulfanyl)methylidene]benzohydrazide, (I), was obtained from an attempt to prepare *S*-benzyl isonicotinoylcarbonohydrazonodithioate (see *Experimental*).

In (I), Fig. 1, the amide is twisted out of the plane through the S_2_C═N atoms with the C1—N1—N2—C16 torsion angle being 139.71 (13)°. A similar twist was found in the structure of (PhCH_2_S)_2_C═ NN(H)C(═ O)C_6_H_4_OMe-4 (Jasinski *et al.*, 2010) but a planar arrangement was observed in the structure of (PhCH_2_S)_2_C═NN(H)C(═O)C_6_H_4_OMe-2 (Singh *et al.*, 2007). The dihedral angle between the phenyl rings is 76.42 (9)°, indicating an almost orthogonal relationship. Each of these rings forms a dihedral angle of 52.96 (8) and 86.46 (8)° with the pyridyl ring.

The crystal packing features supramolecular chains sustained by N—H···O hydrogen bonds, Table 1, and propagated by glide symmetry along the *c* axis, Fig. 2. Chains are consolidated into a three-dimensional architecture by C—H···O and C—H···N interactions, Fig. 3 and Table 1.

### Experimental

The procedure to synthesize *S*-benzyldithiocarbazate (Ali & Tarafder, 1977) was adapted to prepare *S*-benzyl isonicotinoylcarbonohydrazono dithioate by replacing hydrazine with its isonicotinic acid derivative. Potassium hydroxide (0.2 mol, 11.2 g) in absolute ethanol (70 ml) was added to a suspension of isonicotinic acid hydrazide (0.2 mol, 27.43 g) in absolute ethanol (700 ml). The pale-yellow solution was kept in an ice-salt bath and carbon disulfide (0.2 mol) was added drop-wise with constant stirring over one hour. Benzylchloride (0.2 mol, 23 ml) was then added drop-wise with vigorous stirring to the pale-orange solution obtained above. The reaction temperature was maintained below 278 K. An unidentified pale-yellow solid (33.84 g) which did not contain any benzyl substituent was filtered from the mixture. The filtrate was kept in a freezer for one week before it was used as replacement for absolute ethanol to repeat the above reaction. The final solution produced dark-yellow blocks of the title compound after storage at 268 K for 5 months. (Yield 16 g; *M*.pt: 369 K).

### Refinement

Carbon-bound H-atoms were placed in calculated positions (C—H = 0.95 to 0.99 Å) and were included in the refinement in the riding model approximation with *U*_iso_(H) set to 1.2*U*_equiv_(C). The amino H-atom was refined with a distance restraint of N—H = 0.88±0.01 Å, and with *U*_iso_(H) = 1.2*U*_eq_(N).

### Crystal data

**Table:**

C_21_H_19_N_3_OS_2_	*F*(000) = 824
*M**_r_* = 393.51	*D*_x_ = 1.309 Mg m^−^^3^
Monoclinic, *P*2_1_/*c*	Cu *K*α radiation, λ = 1.54180 Å
Hall symbol: -P 2ybc	Cell parameters from 17900 reflections
*a* = 11.2593 (4) Å	θ = 4–71°
*b* = 21.2182 (7) Å	µ = 2.54 mm^−^^1^
*c* = 8.6041 (3) Å	*T* = 150 K
β = 103.678 (3)°	Block, dark yellow
*V* = 1997.24 (12) Å^3^	0.50 × 0.36 × 0.16 mm
*Z* = 4	

### Data collection

**Table:**

Agilent Xcaliber Eos Gemini diffractometer	3867 independent reflections
Radiation source: fine-focus sealed tube	3715 reflections with *I* > 2σ(*I*)
Graphite monochromator	*R*_int_ = 0.031
Detector resolution: 16.1952 pixels mm^-1^	θ_max_ = 71.4°, θ_min_ = 4.0°
ω/2θ scans	*h* = −13→13
Absorption correction: multi-scan (*CrysAlis PRO*; Agilent, 2011)	*k* = −24→26
*T*_min_ = 0.42, *T*_max_ = 0.67	*l* = −10→10
38338 measured reflections	

### Refinement

**Table:**

Refinement on *F*^2^	Primary atom site location: structure-invariant direct methods
Least-squares matrix: full	Secondary atom site location: difference Fourier map
*R*[*F*^2^ > 2σ(*F*^2^)] = 0.035	Hydrogen site location: inferred from neighbouring sites
*wR*(*F*^2^) = 0.097	H atoms treated by a mixture of independent and constrained refinement
*S* = 1.03	*w* = 1/[σ^2^(*F*_o_^2^) + (0.0631*P*)^2^ + 0.6548*P*] where *P* = (*F*_o_^2^ + 2*F*_c_^2^)/3
3867 reflections	(Δ/σ)_max_ = 0.001
247 parameters	Δρ_max_ = 0.22 e Å^−^^3^
0 restraints	Δρ_min_ = −0.32 e Å^−^^3^

### Special details

**Table:**

Geometry. All s.u.'s (except the s.u. in the dihedral angle between two l.s. planes) are estimated using the full covariance matrix. The cell s.u.'s are taken into account individually in the estimation of s.u.'s in distances, angles and torsion angles; correlations between s.u.'s in cell parameters are only used when they are defined by crystal symmetry. An approximate (isotropic) treatment of cell s.u.'s is used for estimating s.u.'s involving l.s. planes.
Refinement. Refinement of *F*^2^ against ALL reflections. The weighted *R*-factor *wR* and goodness of fit *S* are based on *F*^2^, conventional *R*-factors *R* are based on *F*, with *F* set to zero for negative *F*^2^. The threshold expression of *F*^2^ > 2σ(*F*^2^) is used only for calculating *R*-factors(gt) *etc*. and is not relevant to the choice of reflections for refinement. *R*-factors based on *F*^2^ are statistically about twice as large as those based on *F*, and *R*- factors based on ALL data will be even larger.

### Fractional atomic coordinates and isotropic or equivalent isotropic displacement parameters (Å^2^)

**Table:**

	*x*	*y*	*z*	*U*_iso_*/*U*_eq_	
S1	0.58342 (3)	0.395419 (15)	0.24637 (4)	0.02644 (11)	
S2	0.37808 (3)	0.403747 (16)	0.41378 (4)	0.03091 (12)	
O1	0.55367 (12)	0.19229 (5)	0.45894 (12)	0.0423 (3)	
N1	0.45622 (11)	0.29676 (5)	0.32219 (13)	0.0266 (2)	
N2	0.53822 (10)	0.26152 (5)	0.25652 (13)	0.0246 (2)	
H2N	0.5455 (15)	0.2700 (8)	0.161 (2)	0.030*	
N3	0.82288 (13)	0.08675 (8)	0.1580 (2)	0.0510 (4)	
C1	0.47186 (11)	0.35652 (7)	0.32465 (14)	0.0240 (3)	
C2	0.58359 (14)	0.47567 (7)	0.32153 (18)	0.0316 (3)	
H2A	0.5016	0.4948	0.2845	0.038*	
H2B	0.6056	0.4758	0.4399	0.038*	
C3	0.67684 (13)	0.51237 (7)	0.25762 (17)	0.0288 (3)	
C4	0.64021 (15)	0.55501 (9)	0.1339 (2)	0.0423 (4)	
H4	0.5556	0.5605	0.0867	0.051*	
C5	0.72615 (19)	0.58997 (10)	0.0779 (2)	0.0525 (5)	
H5	0.7001	0.6193	−0.0068	0.063*	
C6	0.84883 (17)	0.58206 (9)	0.1453 (2)	0.0465 (4)	
H6	0.9076	0.6057	0.1069	0.056*	
C7	0.88623 (15)	0.53955 (8)	0.2688 (2)	0.0421 (4)	
H7	0.9709	0.5341	0.3155	0.051*	
C8	0.80055 (14)	0.50489 (7)	0.32481 (19)	0.0351 (3)	
H8	0.8269	0.4758	0.4099	0.042*	
C9	0.27606 (13)	0.34592 (7)	0.47123 (17)	0.0297 (3)	
H9A	0.3219	0.3063	0.5034	0.036*	
H9B	0.2487	0.3620	0.5651	0.036*	
C10	0.16574 (13)	0.33186 (7)	0.33872 (17)	0.0308 (3)	
C11	0.15710 (15)	0.27626 (9)	0.2525 (2)	0.0469 (4)	
H11	0.2216	0.2463	0.2766	0.056*	
C12	0.05412 (18)	0.26418 (11)	0.1306 (3)	0.0620 (6)	
H12	0.0489	0.2261	0.0712	0.074*	
C13	−0.04034 (18)	0.30698 (12)	0.0955 (2)	0.0582 (5)	
H13	−0.1102	0.2986	0.0116	0.070*	
C14	−0.03337 (18)	0.36186 (10)	0.1820 (2)	0.0555 (5)	
H14	−0.0989	0.3912	0.1591	0.067*	
C15	0.06947 (16)	0.37429 (8)	0.3028 (2)	0.0441 (4)	
H15	0.0741	0.4124	0.3618	0.053*	
C16	0.57822 (13)	0.20778 (6)	0.33232 (15)	0.0262 (3)	
C17	0.66171 (12)	0.16710 (7)	0.26297 (16)	0.0277 (3)	
C18	0.72075 (14)	0.18668 (8)	0.14762 (18)	0.0352 (3)	
H18	0.7077	0.2277	0.1026	0.042*	
C19	0.79978 (15)	0.14459 (10)	0.0994 (2)	0.0475 (4)	
H19	0.8397	0.1581	0.0195	0.057*	
C20	0.76652 (16)	0.06886 (9)	0.2706 (2)	0.0489 (4)	
H20	0.7824	0.0278	0.3144	0.059*	
C21	0.68633 (15)	0.10680 (7)	0.3272 (2)	0.0381 (4)	
H21	0.6487	0.0921	0.4082	0.046*	

### Atomic displacement parameters (Å^2^)

**Table:**

	*U*^11^	*U*^22^	*U*^33^	*U*^12^	*U*^13^	*U*^23^
S1	0.02696 (19)	0.02388 (19)	0.03094 (19)	−0.00467 (12)	0.01177 (14)	−0.00630 (12)
S2	0.0295 (2)	0.02387 (19)	0.0439 (2)	−0.00234 (12)	0.01763 (16)	−0.00832 (13)
O1	0.0760 (9)	0.0293 (6)	0.0282 (5)	0.0116 (5)	0.0256 (5)	0.0044 (4)
N1	0.0300 (6)	0.0239 (6)	0.0292 (6)	0.0011 (4)	0.0134 (5)	−0.0024 (4)
N2	0.0309 (6)	0.0230 (6)	0.0232 (5)	0.0012 (4)	0.0129 (4)	−0.0005 (4)
N3	0.0308 (7)	0.0560 (10)	0.0626 (10)	0.0119 (7)	0.0037 (7)	−0.0243 (8)
C1	0.0233 (6)	0.0253 (7)	0.0238 (6)	−0.0008 (5)	0.0067 (5)	−0.0035 (5)
C2	0.0332 (7)	0.0232 (7)	0.0420 (8)	−0.0061 (5)	0.0159 (6)	−0.0090 (6)
C3	0.0312 (7)	0.0242 (7)	0.0328 (7)	−0.0057 (5)	0.0115 (6)	−0.0065 (5)
C4	0.0343 (8)	0.0482 (10)	0.0408 (8)	−0.0068 (7)	0.0017 (6)	0.0066 (7)
C5	0.0559 (11)	0.0585 (12)	0.0407 (9)	−0.0109 (9)	0.0067 (8)	0.0181 (8)
C6	0.0450 (9)	0.0502 (10)	0.0493 (9)	−0.0164 (8)	0.0213 (8)	0.0037 (8)
C7	0.0292 (8)	0.0413 (9)	0.0567 (10)	−0.0062 (7)	0.0123 (7)	0.0017 (7)
C8	0.0325 (8)	0.0301 (8)	0.0432 (8)	−0.0020 (6)	0.0096 (6)	0.0034 (6)
C9	0.0297 (7)	0.0292 (7)	0.0340 (7)	−0.0023 (5)	0.0153 (6)	−0.0026 (6)
C10	0.0312 (7)	0.0316 (7)	0.0339 (7)	−0.0053 (6)	0.0166 (6)	−0.0028 (6)
C11	0.0328 (8)	0.0466 (10)	0.0657 (11)	−0.0076 (7)	0.0207 (8)	−0.0230 (9)
C12	0.0445 (10)	0.0750 (14)	0.0714 (13)	−0.0194 (10)	0.0237 (9)	−0.0421 (11)
C13	0.0388 (10)	0.0868 (15)	0.0471 (10)	−0.0165 (10)	0.0069 (8)	−0.0133 (10)
C14	0.0430 (10)	0.0600 (12)	0.0576 (11)	0.0036 (9)	0.0004 (8)	0.0024 (9)
C15	0.0433 (9)	0.0379 (9)	0.0487 (9)	0.0024 (7)	0.0058 (7)	−0.0035 (7)
C16	0.0341 (7)	0.0230 (7)	0.0217 (6)	−0.0012 (5)	0.0071 (5)	−0.0037 (5)
C17	0.0261 (7)	0.0296 (7)	0.0246 (6)	0.0005 (5)	0.0006 (5)	−0.0084 (5)
C18	0.0296 (7)	0.0448 (9)	0.0314 (7)	0.0052 (6)	0.0073 (6)	−0.0037 (6)
C19	0.0301 (8)	0.0687 (12)	0.0451 (9)	0.0065 (8)	0.0116 (7)	−0.0148 (9)
C20	0.0382 (9)	0.0370 (9)	0.0662 (11)	0.0098 (7)	0.0018 (8)	−0.0138 (8)
C21	0.0374 (8)	0.0283 (8)	0.0465 (9)	0.0024 (6)	0.0059 (7)	−0.0062 (6)

### Geometric parameters (Å, º)

**Table:**

S1—C1	1.7643 (13)	C8—H8	0.9500
S1—C2	1.8213 (14)	C9—C10	1.504 (2)
S2—C1	1.7579 (13)	C9—H9A	0.9900
S2—C9	1.8270 (14)	C9—H9B	0.9900
O1—C16	1.2304 (17)	C10—C11	1.385 (2)
N1—C1	1.2795 (18)	C10—C15	1.387 (2)
N1—N2	1.4063 (15)	C11—C12	1.391 (3)
N2—C16	1.3376 (18)	C11—H11	0.9500
N2—H2N	0.867 (18)	C12—C13	1.377 (3)
N3—C20	1.333 (3)	C12—H12	0.9500
N3—C19	1.329 (3)	C13—C14	1.374 (3)
C2—C3	1.5113 (19)	C13—H13	0.9500
C2—H2A	0.9900	C14—C15	1.386 (3)
C2—H2B	0.9900	C14—H14	0.9500
C3—C8	1.385 (2)	C15—H15	0.9500
C3—C4	1.384 (2)	C16—C17	1.5001 (18)
C4—C5	1.392 (2)	C17—C18	1.383 (2)
C4—H4	0.9500	C17—C21	1.395 (2)
C5—C6	1.376 (3)	C18—C19	1.391 (2)
C5—H5	0.9500	C18—H18	0.9500
C6—C7	1.382 (3)	C19—H19	0.9500
C6—H6	0.9500	C20—C21	1.381 (2)
C7—C8	1.387 (2)	C20—H20	0.9500
C7—H7	0.9500	C21—H21	0.9500
			
C1—S1—C2	104.06 (6)	S2—C9—H9B	109.0
C1—S2—C9	102.48 (7)	H9A—C9—H9B	107.8
C1—N1—N2	115.64 (11)	C11—C10—C15	118.79 (15)
C16—N2—N1	115.76 (11)	C11—C10—C9	121.06 (14)
C16—N2—H2N	123.0 (12)	C15—C10—C9	120.14 (14)
N1—N2—H2N	119.4 (11)	C10—C11—C12	120.09 (18)
C20—N3—C19	117.05 (15)	C10—C11—H11	120.0
N1—C1—S2	118.53 (10)	C12—C11—H11	120.0
N1—C1—S1	124.39 (10)	C13—C12—C11	120.47 (18)
S2—C1—S1	117.07 (8)	C13—C12—H12	119.8
C3—C2—S1	107.16 (9)	C11—C12—H12	119.8
C3—C2—H2A	110.3	C12—C13—C14	119.84 (18)
S1—C2—H2A	110.3	C12—C13—H13	120.1
C3—C2—H2B	110.3	C14—C13—H13	120.1
S1—C2—H2B	110.3	C13—C14—C15	119.87 (19)
H2A—C2—H2B	108.5	C13—C14—H14	120.1
C8—C3—C4	118.91 (14)	C15—C14—H14	120.1
C8—C3—C2	120.38 (13)	C14—C15—C10	120.92 (17)
C4—C3—C2	120.69 (13)	C14—C15—H15	119.5
C3—C4—C5	120.62 (16)	C10—C15—H15	119.5
C3—C4—H4	119.7	O1—C16—N2	122.64 (13)
C5—C4—H4	119.7	O1—C16—C17	119.44 (12)
C6—C5—C4	119.99 (16)	N2—C16—C17	117.84 (12)
C6—C5—H5	120.0	C18—C17—C21	118.39 (14)
C4—C5—H5	120.0	C18—C17—C16	124.44 (13)
C5—C6—C7	119.77 (15)	C21—C17—C16	117.05 (13)
C5—C6—H6	120.1	C17—C18—C19	118.11 (16)
C7—C6—H6	120.1	C17—C18—H18	120.9
C6—C7—C8	120.20 (16)	C19—C18—H18	120.9
C6—C7—H7	119.9	N3—C19—C18	124.13 (17)
C8—C7—H7	119.9	N3—C19—H19	117.9
C3—C8—C7	120.51 (15)	C18—C19—H19	117.9
C3—C8—H8	119.7	N3—C20—C21	123.60 (18)
C7—C8—H8	119.7	N3—C20—H20	118.2
C10—C9—S2	112.79 (10)	C21—C20—H20	118.2
C10—C9—H9A	109.0	C20—C21—C17	118.70 (17)
S2—C9—H9A	109.0	C20—C21—H21	120.7
C10—C9—H9B	109.0	C17—C21—H21	120.6
			
C1—N1—N2—C16	139.71 (13)	C9—C10—C11—C12	179.87 (16)
N2—N1—C1—S2	−176.89 (9)	C10—C11—C12—C13	−0.5 (3)
N2—N1—C1—S1	2.25 (17)	C11—C12—C13—C14	−0.5 (3)
C9—S2—C1—N1	−2.38 (12)	C12—C13—C14—C15	0.9 (3)
C9—S2—C1—S1	178.42 (7)	C13—C14—C15—C10	−0.4 (3)
C2—S1—C1—N1	−168.24 (12)	C11—C10—C15—C14	−0.6 (3)
C2—S1—C1—S2	10.91 (9)	C9—C10—C15—C14	−179.43 (16)
C1—S1—C2—C3	−179.46 (10)	N1—N2—C16—O1	−5.9 (2)
S1—C2—C3—C8	−76.56 (15)	N1—N2—C16—C17	177.46 (11)
S1—C2—C3—C4	105.00 (15)	O1—C16—C17—C18	−162.44 (15)
C8—C3—C4—C5	−0.1 (3)	N2—C16—C17—C18	14.3 (2)
C2—C3—C4—C5	178.34 (16)	O1—C16—C17—C21	13.6 (2)
C3—C4—C5—C6	0.3 (3)	N2—C16—C17—C21	−169.68 (13)
C4—C5—C6—C7	−0.3 (3)	C21—C17—C18—C19	1.3 (2)
C5—C6—C7—C8	0.1 (3)	C16—C17—C18—C19	177.25 (13)
C4—C3—C8—C7	−0.1 (2)	C20—N3—C19—C18	−0.4 (3)
C2—C3—C8—C7	−178.54 (14)	C17—C18—C19—N3	−0.5 (2)
C6—C7—C8—C3	0.1 (3)	C19—N3—C20—C21	0.4 (3)
C1—S2—C9—C10	−86.57 (11)	N3—C20—C21—C17	0.4 (3)
S2—C9—C10—C11	104.40 (15)	C18—C17—C21—C20	−1.3 (2)
S2—C9—C10—C15	−76.77 (16)	C16—C17—C21—C20	−177.54 (14)
C15—C10—C11—C12	1.0 (3)		

### Hydrogen-bond geometry (Å, º)

**Table:**

*D*—H···*A*	*D*—H	H···*A*	*D*···*A*	*D*—H···*A*
N2—H2*N*···O1^i^	0.864 (17)	1.936 (17)	2.7852 (15)	167.4 (16)
C7—H7···N3^ii^	0.95	2.54	3.339 (2)	142
C8—H8···N3^iii^	0.95	2.52	3.424 (2)	158
C18—H18···O1^i^	0.95	2.53	3.365 (2)	147

Symmetry codes: (i) *x*, −*y*+1/2, *z*−1/2; (ii) −*x*+2, *y*+1/2, −*z*+1/2; (iii) *x*, −*y*+1/2, *z*+1/2.
