# *N*′-[Bis(benzyl­sulfan­yl)methyl­idene]benzo­hydrazide. Corrigendum

**DOI:** 10.1107/S2056989016015644

**Published:** 2016-10-28

**Authors:** Shahedeh Tayamon, Thahira Begum S. A. Ravoof, Mohamed Ibrahim Mohamed Tahir, Karen A. Crouse, Edward R. T. Tiekink

**Affiliations:** a Department of Chemistry, Universiti Putra Malaysia, 43400 Serdang, Malaysia; b Department of Chemistry, University of Malaya, 50603 Kuala Lumpur, Malaysia

## Abstract

In the paper by Tayamon *et al.* [*Acta Cryst.* (2012), E**68**, o1640–o1641], the chemical name in the title is incorrect.

## Abstract 2

Erratum to *Acta Cryst.* (2012), E**68**, o1640–o1641.

In the paper by Tayamon *et al.* (2012 ▸), the chemical name was given incorrectly in the title. The correct title should be ‘*N*′-[bis(benzylsulfanyl)methylidene]pyridine-4-carbohydra­zide’. The scheme and chemical data are correct as given in the original article.

## References

[bb1] Tayamon, S., Ravoof, T. B. S. A., Tahir, M. I. M., Crouse, K. A. & Tiekink, E. R. T. (2012). *Acta Cryst.* E**68**, o1640–o1641. 10.1107/S1600536812019472 PMC3379243 22719441

